# Increased optic nerve-region [^18^F]FDG uptake in clinically isolated polymyalgia rheumatica: an exploratory PET/CT study within the GCA-PMR spectrum

**DOI:** 10.3389/fimmu.2026.1869837

**Published:** 2026-07-03

**Authors:** Sebastian E. Serfling, Marc Schmalzing, Yingjun Zhi, Patrick-Pascal Strunz, Hannah Labinsky, Philipp E. Hartrampf, Andreas K. Buck, Thorsten A. Bley, Konstanze V. Guggenberger, Rudolf A. Werner, Michael Gernert

**Affiliations:** 1 Department of Nuclear Medicine, University Hospital Würzburg, Würzburg, Germany; 2 Department of Medicine II, Rheumatology and Clinical Immunology, University Hospital Würzburg, Würzburg, Germany; 3 Department of Otorhinolaryngology, Head and Neck Surgery, University Hospital, Würzburg, Germany; 4 Department of Diagnostic and Interventional Radiology, University Hospital Würzburg, Würzburg, Germany; 5 Department of Diagnostic and Interventional Neuroradiology, University Hospital Würzburg, Würzburg, Germany; 6 Department of Nuclear Medicine, Ludwig-Maximilians-Universität (LMU) University Hospital, Ludwig-Maximilians-Universität (LMU) Munich, Munich, Germany

**Keywords:** [^18^F]FDG PET/CT, GCA-PMR spectrum disease, giant cell arteritis, optic nerve-region uptake, polymyalgia rheumatica

## Abstract

**Objective:**

Polymyalgia rheumatica (PMR) and giant cell arteritis (GCA) are considered part of a shared disease spectrum. While orbital inflammatory changes have been described in GCA, the presence of increased optic nerve-region metabolic activity in PMR remains unclear. This study aimed to evaluate canalicular optic nerve-region [^18^F]FDG uptake in patients with clinically isolated PMR.

**Methods:**

In this retrospective single-center study, PMR patients (without signs and symptoms of GCA), who underwent [^18^F]FDG PET/CT were included. [^18^F]FDG uptake in the canalicular optic nerve region was quantified using SUV_max_, SUV_mean_ and target-to-background ratios (TBR). Patients with unremarkable [^18^F]FDG PET/CT served as controls, and patients with GCA were included for comparison.

**Results:**

PMR patients (*n* = 18) showed significantly higher optic nerve-region [^18^F]FDG uptake compared to controls (median TBR 5.5 [IQR 2.8 – 8.9] vs 2.2 [1.9 – 2.7], *p* = 0.0006). Patients without immunosuppressive therapy demonstrated higher uptake than treated patients. Notably, untreated PMR patients showed canalicular optic nerve-region uptake values comparable to those observed in active GCA (7.3 [5.9 – 9.8] vs 8.0 [5.2 – 10.0], *p* = 0.9799). No PMR patient reported visual symptoms.

**Conclusion:**

Patients with clinically isolated PMR showed increased [^18^F]FDG uptake in the canalicular optic nerve region compared with controls, particularly in the absence of immunosuppressive therapy. Uptake values in untreated PMR overlapped with those observed in active GCA. These exploratory findings are compatible with subclinical inflammatory involvement within the GCA-PMR spectrum, but subclinical cranial GCA and non-neural sources of FDG uptake cannot be excluded. Larger prospective studies are required.

## Introduction

Polymyalgia rheumatica (PMR) and giant cell arteritis (GCA) are widely considered to represent a spectrum of disease (GCA-PMR spectrum disease, GPSD) (1–3) as a relevant portion of PMR patients has vasculitis (the prevalence of subclinical vasculitis ranges from 9 – 29%) (4, 5) and a relevant portion of GCA patients has signs and symptoms of PMR (about 42% at GCA diagnosis (6)). A feared complication of GCA is arteritic anterior ischemic optic neuropathy (AAION) (7), which does not occur in isolated PMR without vasculitis. Inflammation of the optic nerve sheath, the arteries of the orbital and the optic nerves itself have been described in GCA (8). Recently, significant inflammation of the optic nerves detected by 2-deoxy-d-[^18^F]fluoro-D-glucose (FDG) positron emission tomography/computed tomography ([^18^F]FDG PET/CT) has been described in GCA patients (9), even if the patients had no visual symptoms. In contrast, optic nerve involvement in PMR has not been systematically investigated so far. Given the concept of a shared disease spectrum, it remains unclear whether similar subclinical inflammatory changes may also be present in PMR in the absence of overt vasculitis.

Therefore, the aim of this exploratory study was to evaluate canalicular optic nerve-region [^18^F]FDG uptake in patients with clinically isolated PMR. We hypothesized that PMR patients may exhibit increased [^18^F]FDG uptake in this region, potentially reflecting subclinical inflammatory involvement within the GCA-PMR spectrum.

## Patients and methods

### Study design and definitions

A single-center retrospective cohort study including PMR patients. Clinical diagnosis of PMR was established by a rheumatologist and confirmed 6 months after initial diagnosis. All patients fulfilled the ACR/EULAR classification criteria from 2012 (without ultrasound) (10) and had undergone a [^18^F]FDG PET/CT scan, mostly due to an unclear inflammatory constellation and to exclude GCA. The patients had no clinical symptoms suggestive of overt vasculitis and no PET/CT evidence of large-vessel vasculitis. Therefore, they were classified as clinically isolated PMR. However, subclinical cranial GCA or subclinical vasculitic involvement cannot be definitively excluded by PET/CT, particularly given the limited sensitivity of PET for cranial arteries.

Patients were stratified according to intake of immunosuppression: Intake of methotrexate ± prednisolone (any dose) or prednisolone monotherapy > 5mg daily were considered as positive intake of immunosuppression (PMR_+IS_). No intake of immunosuppressive agents or a daily prednisolone dose ≤ 5mg were considered as no immunosuppression (PMR_noIS_).

Definitions of active and inactive disease in the comparison group of GCA patients were described previously (9). Approval by the local ethics committee was not mandatory according to German law as this retrospective study included only clinical routine data. All data were generated in compliance with the declaration of Helsinki.

### [^18^F]FDG PET/CT

All patients fasted for at least 6 hours prior to imaging, while water intake was permitted. Blood glucose levels were measured immediately before tracer injection, and imaging was performed only if glucose levels were ≤ 180 mg/dL.

[^18^F]FDG was administered intravenously at a dose of approximately 3 MBq/kg body weight, corresponding to a mean activity of 256 ± 55 MBq (median: 248 MBq). Image acquisition was initiated after a standardized uptake period of approximately 60 minutes.

#### PET/CT acquisition and reconstruction

Imaging was performed on a hybrid PET/CT system (Biograph mCT 128 and mCT 64 Flow edge, Siemens Healthineers). Whole-body PET data were acquired from the skull base to mid-thigh in 3D mode with an acquisition time of 3 minutes per bed position.

All standard corrections, including attenuation, scatter, random coincidences, decay, and dead time, were applied automatically.

PET images were reconstructed using an ordered-subset expectation maximization (OSEM) algorithm incorporating time-of-flight and point-spread-function modeling when available. Reconstruction parameters included a matrix size of 256 × 256, a slice thickness of 2–3 mm, and a Gaussian post-reconstruction filter (4 mm FWHM).

Standardized uptake values (SUV_max_ and SUV_mean_) were calculated using body-weight normalization. Cross-calibration between the PET/CT system and the dose calibrator was performed according to institutional quality assurance protocols.

#### CT protocol

A low-dose CT scan was acquired for attenuation correction and anatomical localization (120 kVp, automated tube current modulation, slice thickness 3 mm, rotation time 0.5 s). Contrast-enhanced diagnostic CT was performed when clinically indicated.

#### Image analysis

PET/CT images were analyzed on axial fused PET/CT images. The canalicular optic nerve-region was identified bilaterally using the CT component for anatomical orientation. ROIs were manually placed in the canalicular optic nerve region across consecutive axial slices and adapted to the visible anatomical extent of the canalicular optic nerve region on CT-fused PET images. A fixed ROI diameter was not used because of the small size of the canalicular optic nerve region and the close proximity of adjacent orbital and vascular structures. ROI placement was standardized by using the canalicular segment as a reproducible anatomical landmark and by applying the same placement strategy in all patients and controls. The canalicular segment was selected because it represents a relatively reproducible anatomical landmark, allows bilateral assessment, and has previously been used for assessment of optic nerve-region uptake in GCA (9). Care was taken to avoid adjacent structures whenever possible.

Quantitative parameters included SUV_max_ and SUV_mean_ for the primary analyses; SUV_peak_ and total lesion glycolysis (TLG; 41% SUV_max_ threshold) were additionally extracted where applicable. SUV_max_ and SUV_mean_ were recorded separately for the left and right canalicular optic nerve region. Since no significant side-to-side differences were observed, the mean of both sides was used for patient-level analyses. Background activity was determined by placing three venous blood-pool ROIs in the superior vena cava. The mean SUV_mean_ of these three measurements was used for target-to-background ratio calculation.

No partial-volume correction was applied. PET/CT datasets were visually reviewed for relevant patient motion or PET/CT misregistration artifacts affecting the optic nerve region. Formal interobserver reproducibility analysis was not performed. Quantitative analysis was performed by experienced board-certified nuclear medicine physicians. Because of the retrospective clinical-routine design, readers were not fully blinded to clinical information.

### Statistical analysis

Wilcoxon signed-rank tests were applied for paired groups and Mann-Whitney U tests for unpaired groups. Spearman’s tests were used to calculate correlations. Differences were considered significant when two tailed *p*-values were ≤ 0.05 (*), ≤ 0.01 (**), or ≤ 0.001 (***). Excel (Microsoft, Redmond, WA) was used to collect the data. Calculations were done with Prism (V11; GraphPad Software, Boston, MA). Figures were grouped with paint.net (V5; dotPDN LLC, Kirkland, WA). Given the exploratory nature of this study and the small sample size, no formal correction for multiple comparisons was applied. Accordingly, *p*-values are reported descriptively and should be interpreted as hypothesis-generating rather than confirmatory.

## Results

### Patients’ characteristics

In this study, 18 patients with PMR were included of whom 6 (33.3%) were female. The median age was 68.5 years (range 51 – 83). The most common reason for performing the [^18^F]FDG PET/CT in PMR was to search for vasculitis The mean CRP-value was 1.8 mg/dl (range 0.5 – 8.1; normal reference range ≤ 0.5mg/dl) and the median erythrocyte sedimentation rate was 18.0 mm/h (range 6 - 91) at the time of the [^18^F]FDG PET/CT. 9 patients (50.0%) did not take immunosuppressive treatments (or a daily prednisolone dose ≤ 5mg) (PMR_noIS_) and had a mean CRP-value of 2.2 mg/dl. 5 took prednisolone monotherapy (with a daily prednisolone dose > 5mg) and 4 took methotrexate (± prednisolone) (PMR_+IS_) and had a mean CRP-value of 1.4mg/dl. The [^18^F]FDG PET/CTs were performed between 2017 and 2025.

**Figure 1:**
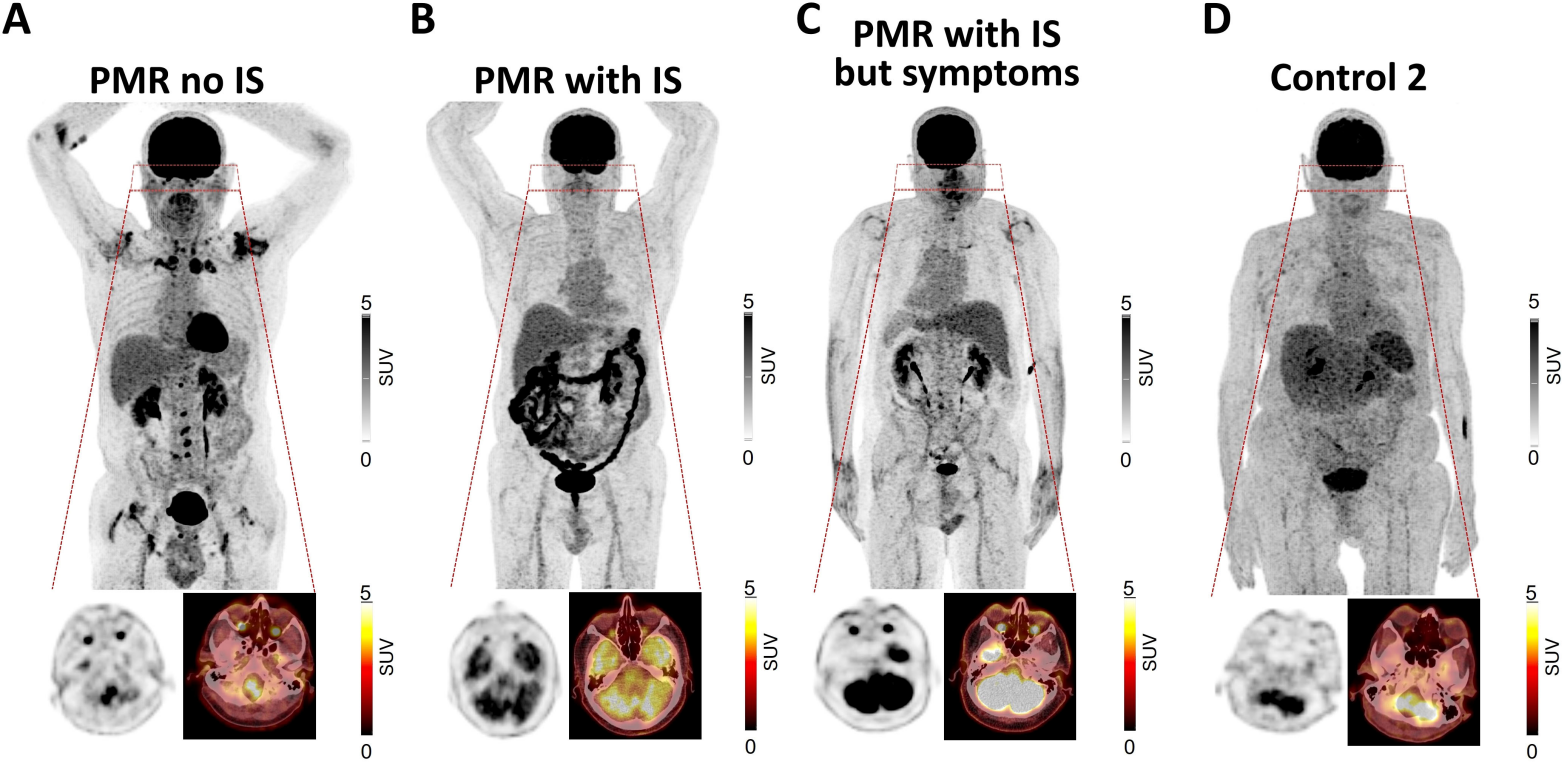
**(A)** Representative increased [^18^F]FDG uptake of both canalicular optic nerve regions in a patient with active polymyalgia rheumatica (PMR) and no immunosuppressive treatment (no IS) without visual symptoms and hypermetabolism of the bilateral shoulder joints as well as the sternoclavicular and acromioclavicular joints, and the bilateral hip joints. In addition, hypermetabolic enthesitis of the pubic symphysis, the spinous processes of the lumbar spine, and the bilateral ischial tuberosities. **(B)** In a patient with known PMR treated with 15 mg prednisolone (PMR_+IS_), [^18^F]FDG PET/CT demonstrated no inflammatory tracer uptake; the optic nerve showed no increased [^18^F]FDG uptake. **(C)** A patient with typical PMR-symptoms despite receiving methotrexate + 5 mg prednisolone. [^18^F]FDG PET/CT demonstrates moderate to intense [^18^F]FDG uptake in the shoulder joints, the hand and finger joints, the bilateral knee joints, and the bilateral upper and lower ankle joints. In addition, increased FDG uptake is observed in the canalicular optic nerve-region. **(D)** Control subject without rheumatologic disease and without immunosuppressive therapy. No increased [^18^F]FDG uptake is observed in the canalicular optic nerve-region.

For comparison, patients with giant cell arteritis (GCA) with active disease, GCA with inactive disease, and a control group 1 (individuals with bronchial carcinoma [BC]) were included. The characteristics of these three groups are published elsewhere (9). Another control group (control 2) was included consisting of 10 people who have received a [^18^F]FDG PET/CT. The most common reason for performing the [^18^F]FDG PET/CT in control group 2 was to search for infection. 2/10 (20.0%) were female, the median age was 74.5 years (range 39 – 89), no one has taken immunosuppressive treatment. **Figure 1** shows representative scans of patients with PMR and of a person from control 2. **Table 1** shows the characteristics of the PMR group and control group 2.

**Table 1:** Characteristics of the study population at the time when [^18^F]FDG PET/CT was performed.

Characteristics^#^	PMR (*n* = 18)	Control 2^§^ (*n* = 10)
Female, *n* (%)	6/18 (33.3)	2/10 (20.0)
Age, median (range), years	68.5 (51 – 83)	74.5 (39 – 89)
Disease duration, median (range), months	1.5 (0 – 13)	na
C-reactive protein, mean (range), mg/dl	1.8 (0.5 – 8.1)	10.0 (0.3 – 22.6)
Erythrocyte sedimentation rate, median (range), mm/h	18.0 (6 – 91)	nd
Inflammatory symptoms		
Unexplained fever	1/18 (5.6)	6/10 (60.0)
B-Symptoms	2/18(11.1)	0/10 (0.0)
Unexplained elevation of C-reactive protein	16/18 (88.9)	8/10 (80.0)
Primary [^18^F]FDG PET/CT question		
Search for infection, *n* (%)	1/18(5.6)	7/10 (70.0)
Search for vasculitis, *n* (%)	13/18 (72.2)	1/10 (10.0)
Search for malignancy, *n* (%)	4/18 (22.2)	2/10 (20.0)
Immunosuppressive treatment		
None, *n* (%)	9/18 (50.0)	10/10 (100.0)
Methotrexate + prednisolone, *n* (%)	4/18 (22.2)	0/10 (0.0)
Prednisolone monotherapy, *n* (%)	5/18 (27.8)	0/10 (0.0)
Prednisolone in those taking, median daily dose (range), mg	15.0 (5 – 50)	na

*[^18^F]FDG PET/CT* 2‐deoxy‐d‐[18F]fluoro‐D‐glucose positron emission tomography/computed tomography; *na* not applicable; *nd* not done; *PMR* polymyalgia rheumatica.

^#^ Characteristics of patients with giant cell arteritis and active disease (GCA active), GCA with inactive disease (GCA inactive), and patients with bronchial carcinoma (control 1) were described elsewhere (9).

^§^ Comprises people who have received a [^18^F]FDG PET/CT to search for an unclear inflammatory focus. Finally, the [^18^F]FDG PET/CT showed no abnormalities.

### Canalicular optic nerve-region [^18^F]FDG uptake does not differ between the left and right side in PMR patients

**Figure 2:**
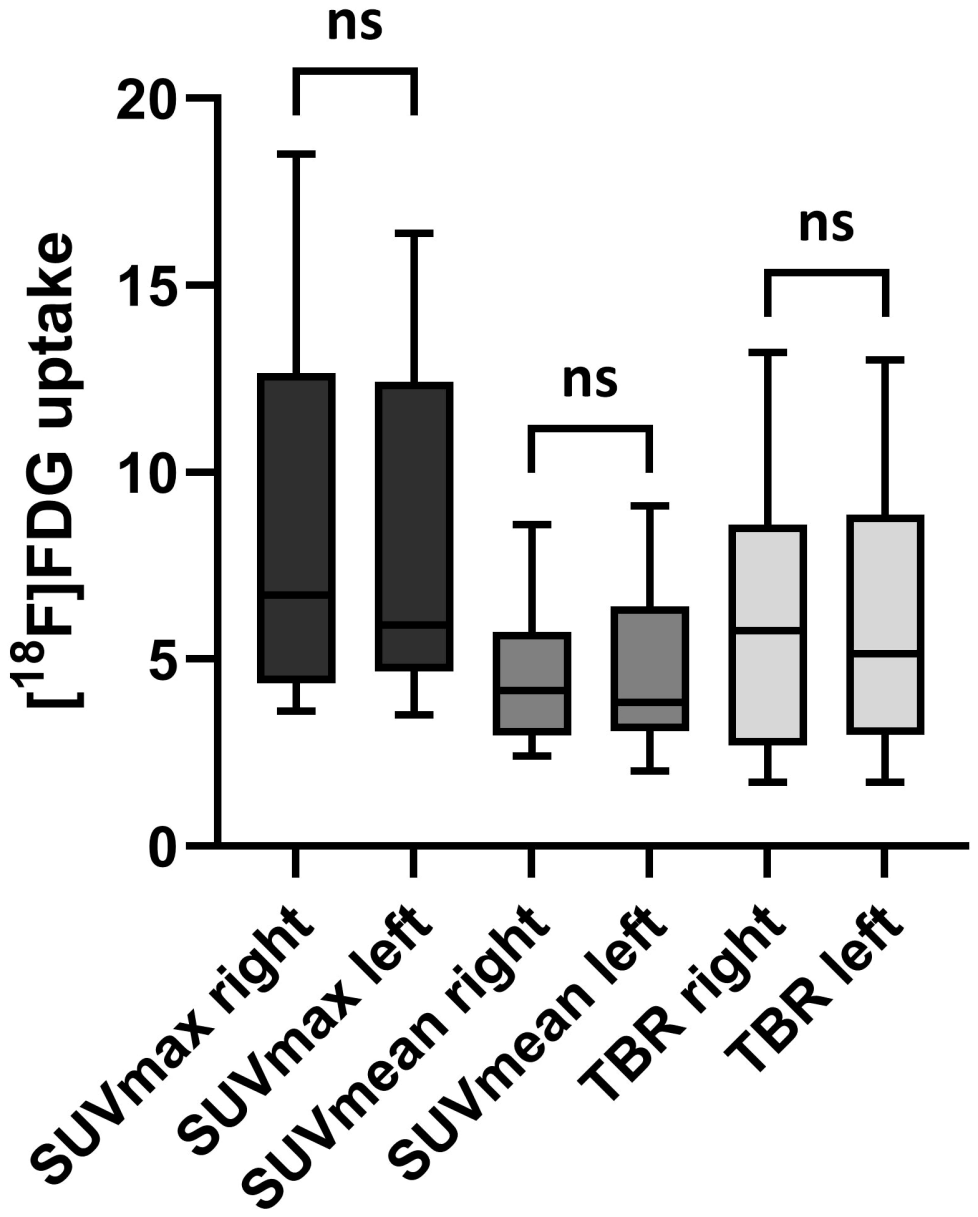
Comparison of [^18^F]FDG uptake of left and right canalicular optic nerve-region in patients with polymyalgia rheumatica. SUV_max_ (black boxes), SUV_mean_ (dark grey boxes), and target-to-background ratio (TBR; light grey boxes). *n* = 18 for each box. Boxes show medians with interquartile ranges, whiskers indicate minimum and maximum; *ns* not significant.

To assess the [^18^F]FDG uptake of the canalicular optic nerve-region, SUV_max_ and SUV_mean_ had to be measured on the left canalicular optic nerve-region and the right canalicular optic nerve-region separately. There was no significant difference in [^18^F]FDG uptake between left and right side (**Figure 2**). Thus, in the following analyses the mean of the left and right canalicular optic nerve-region was used.

### PMR patients receiving immunosuppressive therapy show lower canalicular optic nerve-region [^18^F]FDG uptake

**Figure 3:**
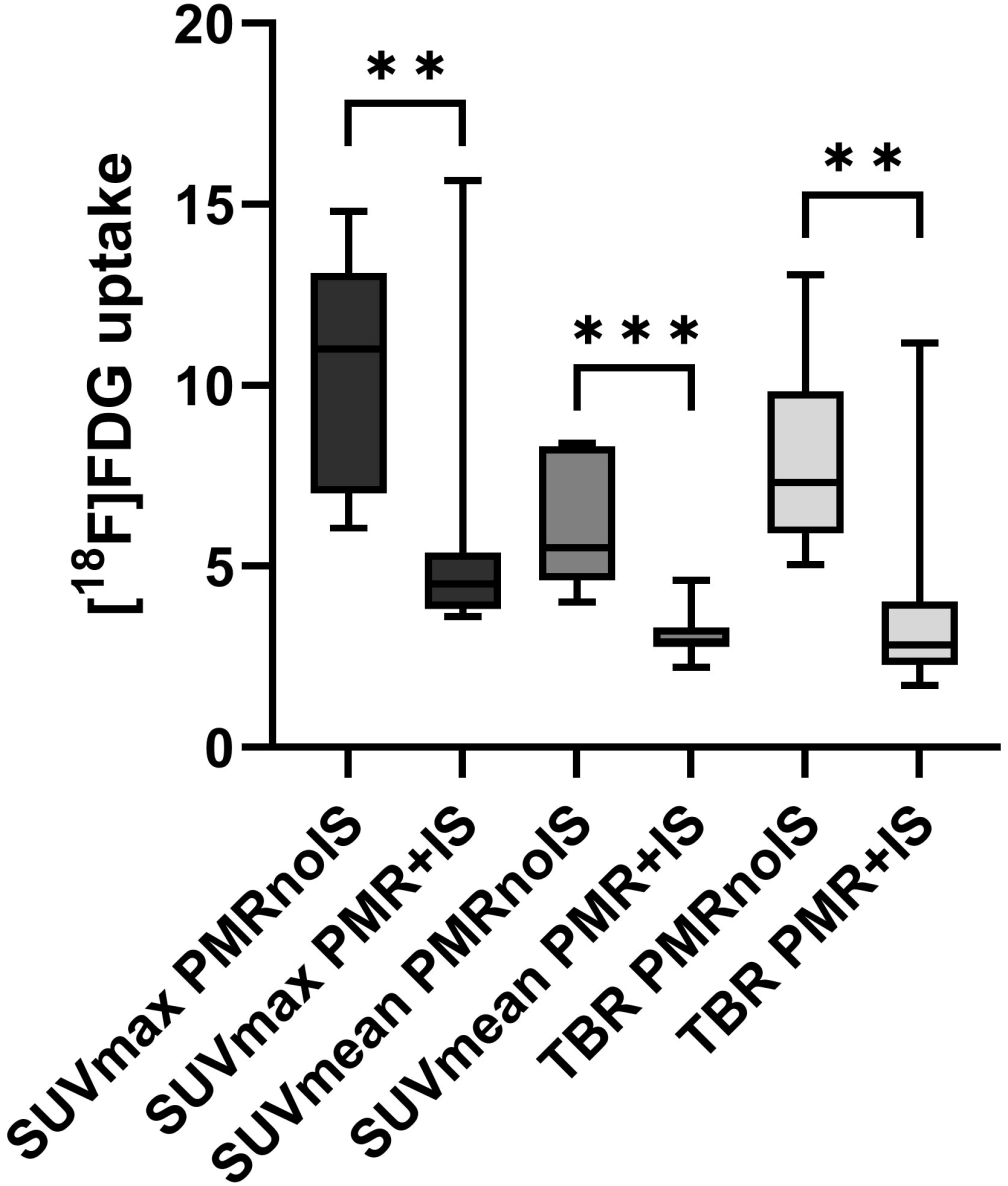
Subgroup analysis of canalicular optic nerve-region [^18^F]FDG uptake comparing PMR patients without immunosuppressive treatment (PMR_noIS_) with patients receiving immunosuppressive treatment (PMR_+IS_). SUV_max_ (black boxes), SUV_mean_ (dark grey boxes), and target-to-background ratio (TBR; light grey boxes). *n* = 9 for each box. Boxes show medians with interquartile ranges, whiskers indicate minimum and maximum; **p ≤ 0.01, ***p ≤ 0.001.

PMR_noIS_ patients showed higher canalicular optic nerve-region [^18^F]FDG uptake than PMR_+IS_ patients (median SUV_max_ 11.0 [IQR 7.0 – 13.1] vs 4.5 [3.9 – 5.4], *p* = 0.0040; median SUV_mean_ 5.5 [4.6 – 8.3] vs 2.9 [2.8 – 3.3], *p* = 0.0002; median TBR 7.3 [5.9 – 9.8] vs 2.8 [2.3 – 4.0], *p* = 0.0028) (**Figure 3**).

### Canalicular optic nerve-region [^18^F]FDG uptake is higher in PMR patients than in controls

**Figure 4:**
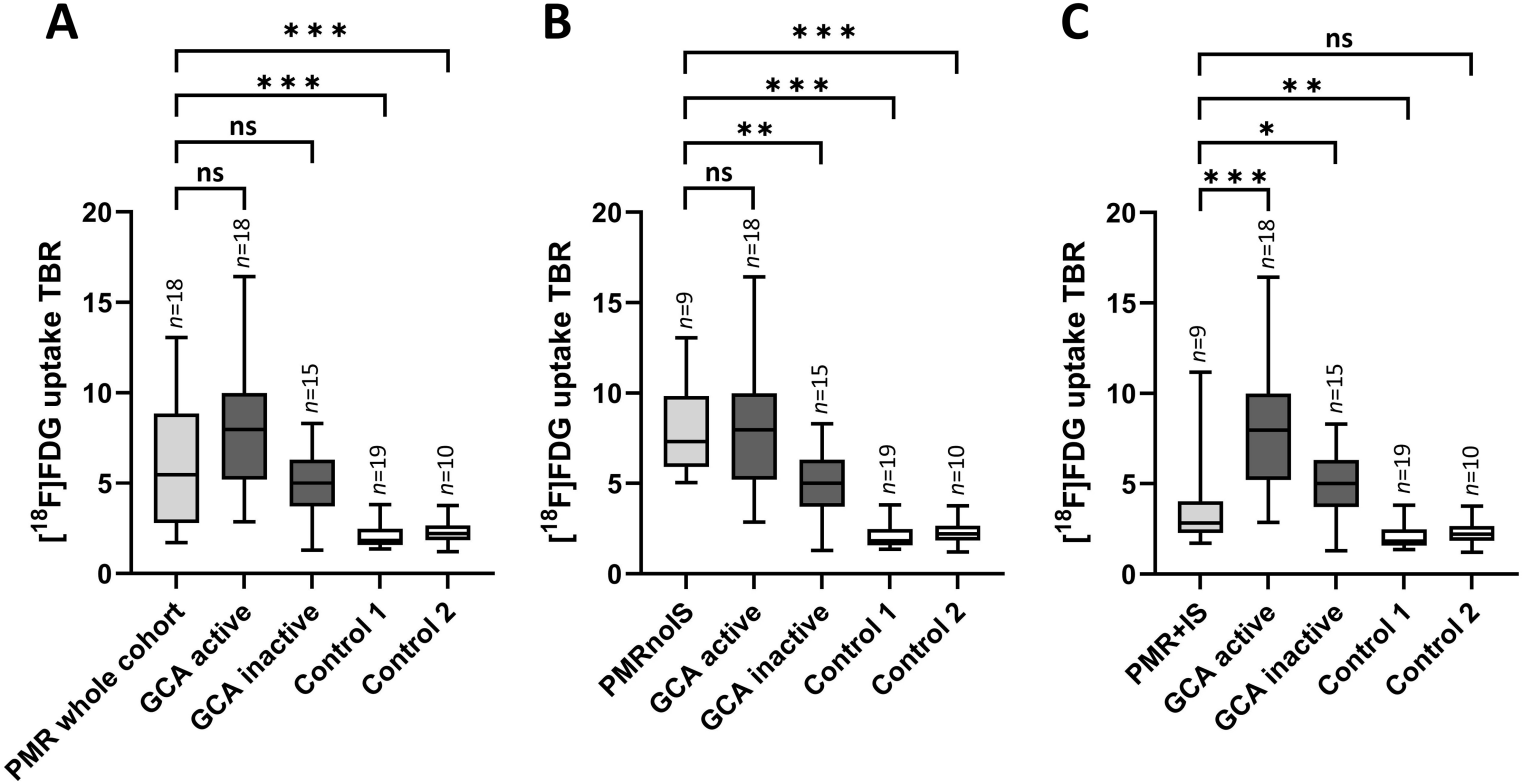
Comparison of [^18^F]FDG uptake (target-to-background ratios, TBR) of the canalicular optic nerve-region between the whole PMR cohort **(A)**, PMR patients without immunosuppressive treatment [PMR_noIS_; **(B)**], and PMR patients with immunosuppressive treatment [PMR_+IS_; **(C)**] to patients with active giant cell arteritis (GCA active), to patients with inactive GCA (GCA inactive), and two control groups (control 1: patients with bronchial carcinoma; control 2: persons with unremarkable [^18^F]FDG PET/CT). Boxes show medians with interquartile ranges, whiskers indicate minimum and maximum; *p ≤ 0.05, **p ≤ 0.01, ***p ≤ 0.001, *ns* not significant. Not shown are *p*-values between GCA active versus GCA inactive/control 1/control 2 (all comparisons *p* ≤ 0.001); GCA inactive versus control 1/control 2 (all comparisons *p* ≤ 0.001); control 1 vs control 2 (*p* = 0.330, not significant).

In the whole PMR group (comprising PMR_+IS_ and PMR_noIS_) a significantly higher canalicular optic nerve-region [^18^F]FDG uptake was detected compared to both control groups (median TBR PMR whole cohort vs control 2: 5.5 [2.8 – 8.9] vs 2.2 [1.9 – 2.7], *p* = 0.0006). No PMR patient had visual symptoms at the time of the [^18^F]FDG PET/CT. However, no standardized ophthalmologic assessment, including fundoscopy, optical coherence tomography, visual field testing, or visual evoked potentials, was available. In the subgroup analyses concerning intake of immunosuppressive treatment, PMR patients with no IS exhibited a higher [^18^F]FDG uptake in the canalicular optic nerve-region compared to both control groups. PMR_+IS_ showed a significantly higher [^18^F]FDG uptake in the canalicular optic nerve-region only compared to control group 1, but not compared to control group 2 (**Figure 4**).

### Canalicular optic nerve-region [^18^F]FDG uptake in PMR compared with GCA

In comparison with GCA, no differences in the [^18^F]FDG uptake of the canalicular optic nerve-regions was detected in the active/not treated state of both entities (PMR_noIS_ vs GCA active: median TBR 7.3 [5.9 – 9.8] vs 8.0 [5.2 – 10.0], *p* = 0.9799). Comparing both entities in the inactive/treated state, a significant lower [^18^F]FDG uptake in the canalicular optic nerve-regions was detected in PMR_+IS_ versus GCA inactive (median TBR 2.8 [2.3 – 4.0] vs 5.0 [3.7 – 6.3], *p* = 0.0203). PMR_noIS_ had significantly higher canalicular optic nerve-region [^18^F]FDG uptake than patients with treated/inactive GCA (*p* = 0.0042) (**Figure 4**).

### Comparison of arterial and canalicular optic nerve-region [^18^F]FDG uptake

**Figure 5:**
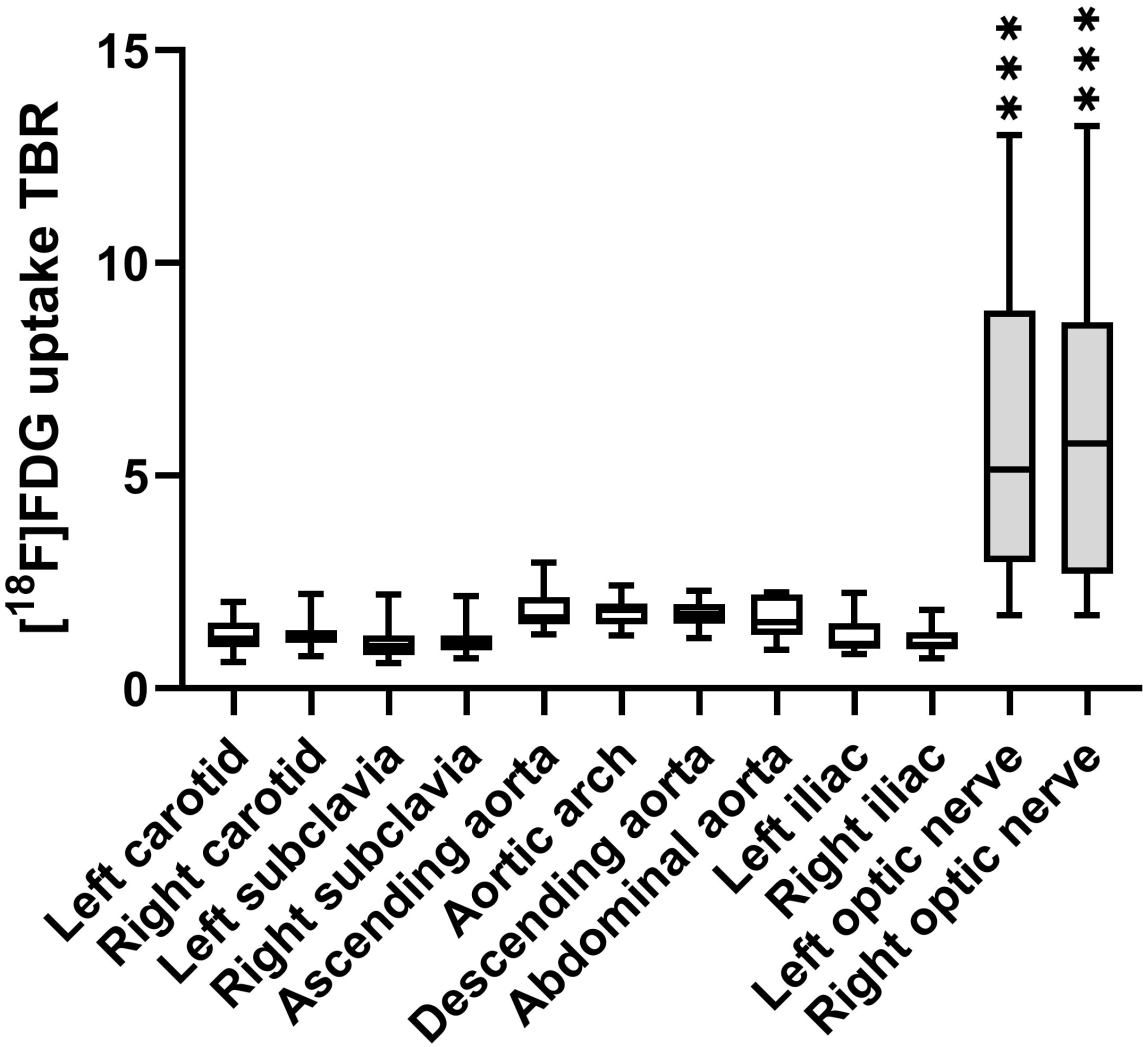
Comparison of canalicular optic nerve-region [^18^F]FDG uptake (target-to-background ratios, TBR) with arterial segment uptake. Boxes show medians with interquartile ranges, whiskers indicate minimum and maximum; ***p ≤ 0.001 between left or right canalicular optic nerve-region and each arterial segment. For each region *n* = 18.

To assess for PET/CT evidence of large-vessel vasculitis, [^18^F]FDG uptake of several arterial segments was evaluated and compared with canalicular optic nerve-region uptake. No relevant [^18^F]FDG uptake was measured in the arteries with a consistent and significant lower uptake in the arteries compared to the canalicular optic nerve-regions (for example median TBR of the aortic arch vs the left ON: 1.9 [1.5 – 2.0] vs 5.1 [3.0 – 8.9], *p* < 0.0001) (**Figure 5**).

### Canalicular optic nerve-region [^18^F]FDG uptake and serologic inflammatory markers in PMR

No correlation was detected between the CRP-value or the ESR with the [^18^F]FDG uptake of the canalicular optic nerve regions (**Supplementary Figure 1**).

## Discussion

In this exploratory study, [^18^F]FDG PET/CT revealed increased metabolic activity in the canalicular optic nerve region in patients with clinically isolated polymyalgia rheumatica. Compared to two independent control groups, PMR patients exhibited significantly higher FDG uptake of the canalicular optic nerve regions. Within the PMR cohort, patients receiving immunosuppressive therapy showed markedly reduced uptake compared to patients without immunosuppressive therapy. This association with treatment is compatible with an inflammation-related component of the PET signal. Notably, none of the PMR patients reported visual symptoms, indicating that increased optic nerve-region uptake may occur in the absence of reported visual symptoms.

Interestingly, patients with untreated PMR showed canalicular optic nerve-region [^18^F]FDG uptake comparable to that observed in patients with active GCA. Given that orbital involvement has rarely been described in PMR and remains insufficiently characterized even in GCA (8, 11), our results point towards a previously underrecognized imaging finding within the GCA-PMR spectrum.

A critical question is whether the observed optic nerve-region uptake reflects clinically isolated PMR or subclinical cranial GCA. Although none of the PMR patients had clinical symptoms suggestive of overt vasculitis and no PET/CT evidence of large-vessel vasculitis was present, subclinical cranial GCA cannot be definitively excluded. This is particularly relevant because PET/CT has limited sensitivity for cranial arteries and subclinical GCA may occur in patients presenting with PMR. Therefore, the present findings should not be interpreted as proof of PMR-specific optic nerve involvement, but rather as exploratory evidence of increased canalicular optic nerve-region uptake within the GCA-PMR spectrum.

In inactive and treated PMR, optic nerve-region uptake was lower than in GCA, which may be compatible with a lower inflammatory burden in PMR than in GCA. Similar to GCA, [^18^F]FDG uptake in the optic nerve region in PMR was significantly higher than vascular uptake. These observations argue against overt large-vessel vasculitis but do not exclude subclinical cranial GCA or small-vessel involvement.

Although neural tissues may exhibit higher physiological glucose metabolism than vascular structures (12), several findings argue against a purely physiological explanation. The significantly higher [^18^F]FDG uptake in the canalicular optic nerve region in PMR patients compared to both control groups and the lower uptake in treated PMR patients are compatible with an inflammation-related component of the signal.

No correlation was observed between systemic inflammatory markers such as C-reactive protein or erythrocyte sedimentation rate and [^18^F]FDG uptake in the canalicular optic nerve regions. This dissociation suggests that canalicular optic nerve-region uptake may reflect a localized inflammatory process that is not adequately reflected by systemic biomarkers. Due to the limited spatial resolution of [^18^F]FDG PET/CT, the exact anatomical correlate of the increased uptake cannot be determined. Possible anatomical sources of the PET signal include the optic nerve sheath, small perivascular structures such as branches of the ophthalmic artery, the vasa nervorum, or the optic nerve itself. All these structures are described to have signs of inflammation on orbital MRIs in GCA patients with the optic nerve sheath being the most common site of inflammation (8, 13). Therefore, the observed uptake should not be equated with isolated optic nerve inflammation, but rather interpreted as increased metabolic activity in the canalicular optic nerve region with an uncertain anatomical substrate. The lower uptake observed under immunosuppressive therapy and the similarity to findings in GCA are compatible with an inflammation-related component, but the exact biological substrate remains uncertain. The use of target-to-background ratios normalized to the superior vena cava improved robustness of quantification by reducing variability related to systemic tracer distribution and blood glucose levels. The inclusion of two independent control groups (patients with bronchial carcinoma from a previous paper (9) and a group consisting of persons having received a [^18^F]FDG PET/CT to find an unclear inflammatory focus) strengthens the validity of our findings. None of these controls took immunosuppressive treatments at the time of the [^18^F]FDG PET/CT. We did not expect a deviation from physiological [^18^F]FDG uptake in the canalicular optic nerve regions in these groups.

This study has several limitations, including its retrospective single-center design and the relatively small cohort size. Another important limitation is the absence of standardized ophthalmologic assessment. Although no PMR patient reported visual symptoms at the time of PET/CT, the absence of symptoms alone does not exclude subtle or subclinical optic neuropathy. Prospective studies should therefore include systematic ophthalmologic evaluation, including fundoscopy, optical coherence tomography, visual field testing and, where appropriate, visual evoked potentials. In addition, the optic nerve represents a small anatomical structure, and partial volume effects may influence quantitative PET measurements despite standardized analysis. No partial-volume correction was applied, and formal interobserver reproducibility analysis was not performed. Because of the retrospective clinical-routine design, readers were not fully blinded to clinical information. PET/CT datasets were visually reviewed for relevant motion or PET/CT misregistration artifacts, but subtle artifacts cannot be entirely excluded. Finally, multiple exploratory comparisons were performed without formal adjustment for multiple testing; therefore, *p*-values should be interpreted descriptively and as hypothesis-generating. Histopathological confirmation of the observed findings is not available. If confirmed in larger prospective studies, assessment of canalicular optic nerve-region [^18^F]FDG uptake may provide an additional imaging biomarker for subclinical inflammation within the GCA–PMR spectrum. This could contribute to improved disease characterization and may have implications for early detection and treatment monitoring.

In conclusion, this exploratory study demonstrates increased [^18^F]FDG uptake in the canalicular optic nerve region in patients with clinically isolated PMR. The overlap with active GCA supports the hypothesis of shared inflammatory features within the GCA-PMR spectrum. However, the findings do not establish PMR-specific optic nerve inflammation, and subclinical cranial GCA cannot be definitively excluded. Prospective studies with larger cohorts, standardized ophthalmologic assessment, and dedicated imaging protocols are required.

## Data Availability

The raw data supporting the conclusions of this article will be made available by the authors, without undue reservation.

## Supplementary material

Supplementary Figure 1 Correlations between inflammatory markers in the blood (C-reactive protein [CRP] and erythrocyte sedimentation rate [ESR]) and [^18^F]FDG uptake [**(A)** and **(D)** SUV_max_, **(B, E)** SUV_mean_, and **(C, F)** target-to-background ratios, TBR] of canalicular optic nerve regions.
