# Supplementary figures and images for: Increased optic nerve-region [18F]FDG uptake in clinically isolated polymyalgia rheumatica: an exploratory PET/CT study within the GCA-PMR spectrum
Source: Front Immunol. 2026 Jul 3;17:1869837. doi: 10.3389/fimmu.2026.1869837 (PMC13375866; doi:10.3389/fimmu.2026.1869837)

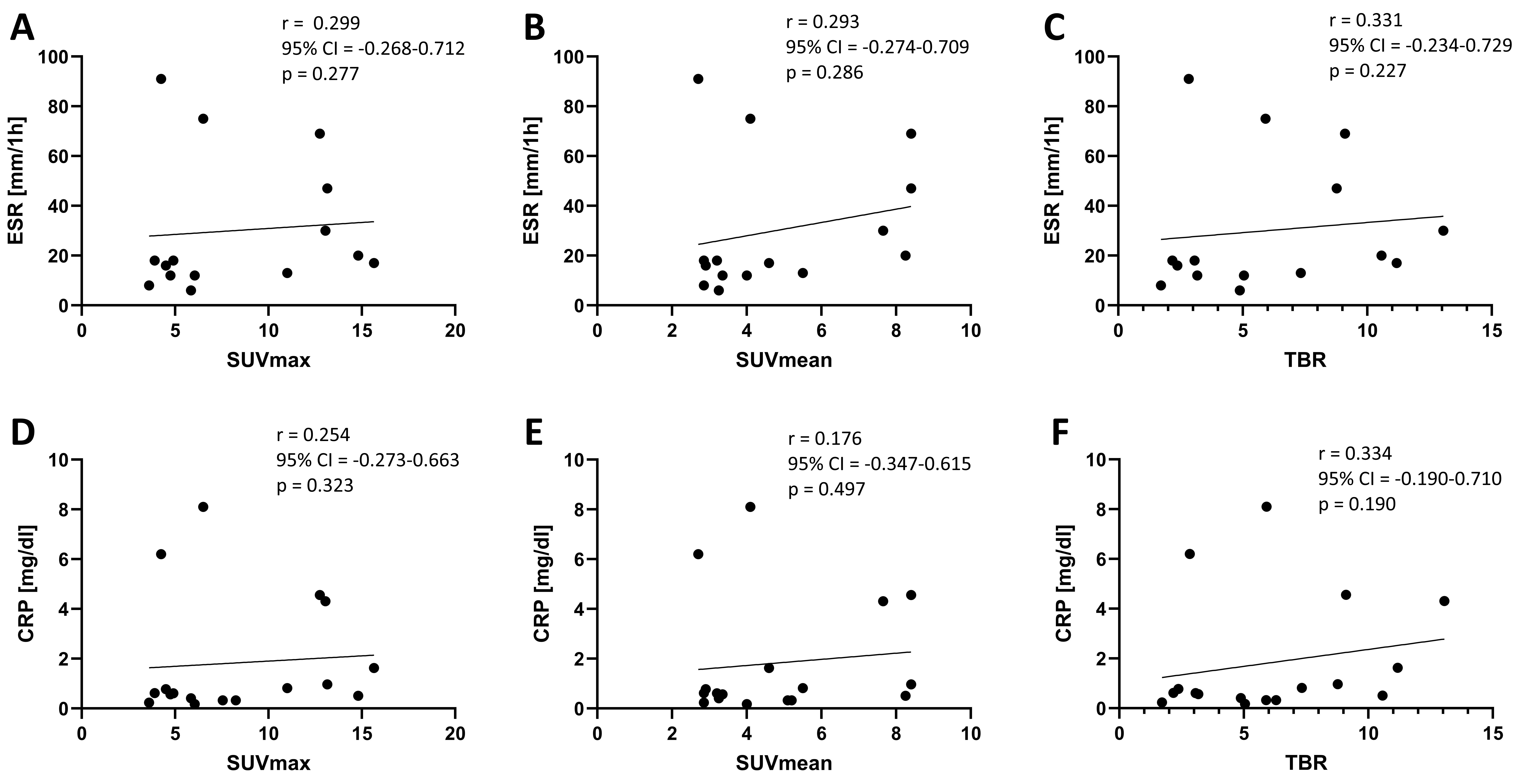

Supplement: Supplementary Figure 1 — Correlations between inflammatory markers in the blood (C-reactive protein [CRP] and erythrocyte sedimentation rate [ESR]) and [18F]FDG uptake [(A) and (D) SUVmax, (B, E) SUVmean, and (C, F) target-to-background ratios, TBR] of canalicular optic nerve regions. [file Image1.jpeg]
